# Educational interventions to improve prescription and dispensing of antibiotics: a systematic review

**DOI:** 10.1186/1471-2458-14-1276

**Published:** 2014-12-15

**Authors:** Fátima Roque, Maria Teresa Herdeiro, Sara Soares, António Teixeira Rodrigues, Luiza Breitenfeld, Adolfo Figueiras

**Affiliations:** Health Sciences Research Centre, University of Beira Interior (Centro de Investigação em Ciências da Saúde – CICS/UBI), Av. Infante D. Henrique, 6200-506 Covilhã, Portugal; Centre for Cell Biology, University of Aveiro (Centro de Biologia Celular – CBC/UA); Campus Universitário de Santiago, 3810-193 Aveiro, Portugal; Research Unit for Inland Development, Polytechnic of Guarda (Unidade de Investigação para o Desenvolvimento do Interior – UDI/IPG), Av. Dr. Francisco Sá Carneiro n°50, 6300-559 Guarda, Portugal; CESPU, Instituto de Investigação e Formação Avançada em Ciências e Tecnologias da Saúde, Rua Central de Gandra, 1317, 4585-116 Gandra, PRD Portugal; Consortium for Biomedical Research in Epidemiology & Public Health (CIBER en Epidemiología y Salud Pública – CIBERESP), University of Santiago de Compostela, R/ de San Francisco, s/n, 15782 Santiago de Compostela, Spain

**Keywords:** Drug resistance microbial, Review, Behavior change, Education medical continuing, Education pharmacy continuing

## Abstract

**Background:**

Excessive and inappropriate antibiotic use contributes to growing antibiotic resistance, an important public-health problem. Strategies must be developed to improve antibiotic-prescribing. Our purpose is to review of educational programs aimed at improving antibiotic-prescribing by physicians and/or antibiotic-dispensing by pharmacists, in both primary-care and hospital settings.

**Methods:**

We conducted a critical systematic search and review of the relevant literature on educational programs aimed at improving antibiotic prescribing and dispensing practice in primary-care and hospital settings, published in January 2001 through December 2011.

**Results:**

We identified 78 studies for analysis, 47 in primary-care and 31 in hospital settings. The studies differed widely in design but mostly reported positive results. Outcomes measured in the reviewed studies were adherence to guidelines, total of antibiotics prescribed, or both, attitudes and behavior related to antibiotic prescribing and quality of pharmacy practice related to antibiotics. Twenty-nine studies (62%) in primary care and twenty-four (78%) in hospital setting reported positive results for all measured outcomes; fourteen studies (30%) in primary care and six (20%) in hospital setting reported positive results for some outcomes and results that were not statistically influenced by the intervention for others; only four studies in primary care and one study in hospital setting failed to report significant post-intervention improvements for all outcomes. Improvement in adherence to guidelines and decrease of total of antibiotics prescribed, after educational interventions, were observed, respectively, in 46% and 41% of all the reviewed studies. Changes in behaviour related to antibiotic-prescribing and improvement in quality of pharmacy practice was observed, respectively, in four studies and one study respectively.

**Conclusion:**

The results show that antibiotic use could be improved by educational interventions, being mostly used multifaceted interventions.

**Electronic supplementary material:**

The online version of this article (doi:10.1186/1471-2458-14-1276) contains supplementary material, which is available to authorized users.

## Background

Antibiotic resistance is an important public-health issue, which is aggravated by the lack of new antimicrobial agents [1, 2]. Inappropriate use of antibiotics is the main factor underlying microbial resistance [3, 4]. Ecological studies in Europe suggest that there is a clear association between extent of antibiotic use and rate of resistance [5]. Excessive and inappropriate use of antibiotics is attributed to misprescription and to self-medication with “leftovers” from previous courses or with antibiotics dispensed in pharmacies without prescription [6, 7]. In countries with a high incidence of self-medication with antibiotics, prescription of antibiotics is also high [7], suggesting that both practices are subject to the same cultural factors [8]. Physicians and pharmacists are the health professionals who exert most influence on patients’ medication-related behavior. Many educational interventions to improve antibiotic-prescribing and/or dispensing have targeted those health professionals. Previous systematic reviews of the topic include Steinman’s [9], which covered reports published prior to 2004 and on interventions directed at physicians. Other more recent reviews [10–13] have targeted specific areas, namely, respiratory tract infections [10, 13], critical care [11], and acute care [12]. Therefore, there has been no general reviews, of the topic, including interventions on physicians a pharmacists to improve antibiotic prescription and dispensing. To close this gap, we carried out a critical review of educational programs aimed at improving antibiotic-prescribing by physicians and/or antibiotic-dispensing by pharmacists, in both primary-care and hospital settings.

## Methods

### Literature search methodology

For review purposes, we conducted a search of the MEDLINE-PubMED scientific database from January 2001 through December 2011. In addition, other papers were located by manual searches targeting journals, particularly those less likely to be indexed, and references cited by papers retrieved.

The search strategy was designed to identify relevant studies addressing antibiotic resistance and the prescribing/dispensing habits of health care providers (physicians and pharmacists) pre- and post-educational interventions. The following search terms and their equivalents were used in PubMed: (“intervention” OR “program” OR “health promotion” OR “education”) AND (“pharmacists” OR “pharmacy” OR “physician” OR “health professionals” OR “clinician” OR “clinic” OR “practitioner” OR “general practitioner” OR “doctor”) AND (“antibiotics” OR “antimicrobial”).

Based on previous reviews [14–17], we apply this selection criteria: (i) language: papers had to be published in English, French, Spanish or Portuguese; (ii) type of intervention: studies had to describe educational interventions; (iii) target population: educational interventions had to target physicians (general practitioners and all specialties) and/or pharmacists (population studies were included only if they also included interventions on pharmacists and/or physicians); and (iv) outcome measures: studies had to measure the effect of educational interventions on the prescribing behavior of physicians and/or dispensing behavior of pharmacists. Insofar as study design was concerned, no inclusion or exclusion criteria were stipulated because our aim was to use quality methodology to conduct a critical review of all published studies.

### Data-extraction

#### Study design

Adapted from Figueiras [18], study designs were classified as follows: (1) before/after study; (2) non-randomized controlled study without cross-contamination control; (3) non-randomized controlled study with cross-contamination control; (4) randomized controlled study without cross-contamination control; and (5) randomized controlled study without cross-contamination control. Where authors reported the different groups as being in workplaces that were geographically far apart, the study was deemed to have cross-contamination control; and where no mention was made of distance between groups or specific implementation of cross-contamination control, the study was deemed to be without such control.

#### Target disease

In cases where studies identified specific diseases in which interventions were made to improve antibiotic use, this was recorded.

#### Type of intervention

Educational interventions include any attempt to persuade physicians to modify their practice performance by communicating clinical information strategies [19] and by communication skills training [13]. Strategies that were purely administrative or applied incentives or coercion were excluded from this definition of educational interventions. In our review, we only included studies that assessed educational interventions. However, in studies in which these types of interventions were associated with others, we extracted data on all strategies. Consequently, interventions were classified into the following categories, adapted from Davis [19] and Figueiras [18]: (1) dissemination of printed/audiovisual educational materials (mailed printed matter; protocols and guidelines; self-instruction materials; drug bulletins); (2) group education, including group-session rounds, conferences, lectures, seminars, and tutorials; (3) feedback of physician prescribing patterns (individually, or including a comparison between these patterns and peer behavior and/or accepted standards), or feedback of patient-specific lists of prescribed medication; (4) individual outreach visits; (5) reminders at the time of prescribing; (6) computer-assisted decision-making systems; (7) formulary control/restrictive formulary process; (8) patient education (pamphlets); (9) patient education (videotapes); (10) workshops on rapid tests/introduction of Rapid Antigen Detection Testing (RADT) in consulting offices; (11) enforcement of regulations; (12) prescription feedback, with recommendations to modify it made by pharmacists and/or infectious-disease physicians; (13) financial incentives.

#### Baseline and follow-up

Under this head, we included the period during which outcomes were measured (baseline, intervention period and follow-up).

#### Analysis

Studies were classified into different categories, namely: (1) comparison of post-intervention values between groups; (2) comparison of pre- and post-intervention values within each group; (3) comparison of pre- and post-intervention values between groups; (4) comparison of follow-up values between groups; (5) comparison of pre-, post, and follow-up values within each group; and, (6) comparison of pre-, post- and follow-up values between groups.

#### Statistical tests

We collected data yielded by statistical tests used to assess the effectiveness of interventions.

#### Results

The results extracted from studies consisted of changes in: total antibiotics prescribed/dispensed (T); choice of appropriate antibiotics/adherence to antibiotic guidance according to guideline algorithms, including dosages and administration routes (Ga); attitudes and behavior (At/Bh); quality of pharmacy practice (Qph).

Study results were classified as: positive (+), if reported as positive or if changes in outcomes measured were statistically significant; partially positive (±), if reported as positive for some variables and negative for others; and negative (−), if reported as negative.

## Results and discussion

### Selection of papers

**Figure 1:**
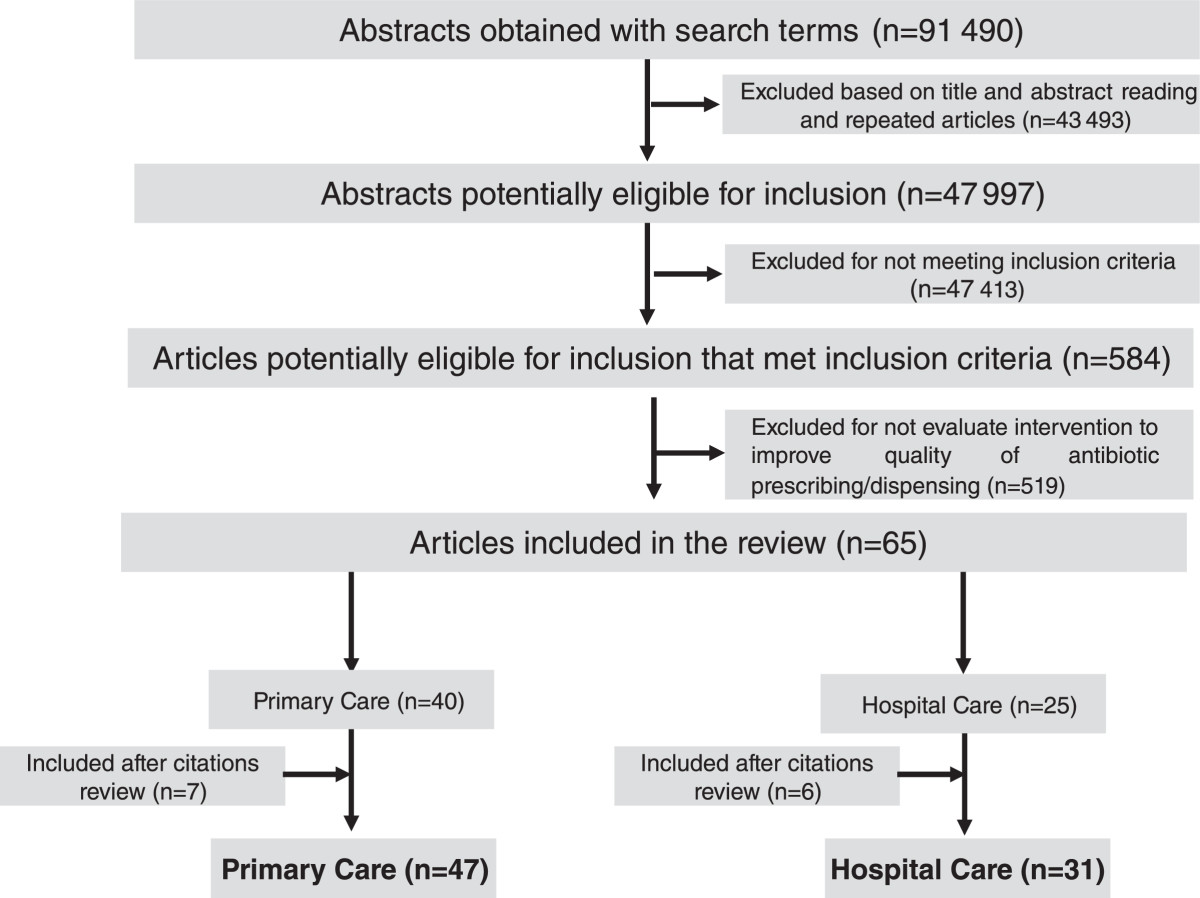
**Identification and inclusion of studies.**

The search yielded a total of 90,350 Abstracts, 47,535 of which were potentially eligible for inclusion. A reading of the titles and abstracts led to an initial selection of 571 papers for full-text analysis; of these, 65 were then selected, made up of 40 primary- and 25 hospital-care studies. After a search of the references cited, 7 papers were added to the primary-care and 6 to the hospital-care studies. A total of 78 papers were included, 47 primary- [20–66] and 31 hospital-care interventions [3, 67–96] (Figure 1)

### Interventions in primary care professionals

In the studies analyzed (Table 1), educational interventions in primary care mainly targeted physicians, and outcomes were assessed by reference to the total antibiotic prescription or appropriate antibiotic prescription rates. Educational interventions in pharmacists occurred in 8 studies [25, 32, 33, 42, 44, 50, 52, 66], though in 6 cases the interventions covered both pharmacists and physicians. In 21 studies [20, 22, 23, 25, 31–33, 35–37, 39, 43, 45, 46, 50–53, 55, 57, 60], the interventions were extended to patients and their caregivers or general population.

**Table 1:** **Studies analyzing educational interventions in health professionals to improve antibiotic use**

*Author (year)*	*Country*	*Allocation unit (a)*	*Intervention population (b)*	*Type of patient*	*Sample size (%) (b), (c)*	*Statistical test*
**Dollman, WB (2005)** [20]	South Australia	PC	GPs, Pa	All	___	Bivariate
**Hrisos, S (2007)** [21]	UK	PC	GPs	___	340 GPs	Multivariate Bivariate
**Hennessy, TW (2002)** [22]	USA (Alaska)	PC	Py, Pa, O	All	3144 Pa	Multivariate Bivariate
**Rubin, MA (2005)** [23]	USA	PC	Py, Pa	All	___	Multivariate
**Naughton, C (2009)** [24]	Ireland	PC	GPs	All	110 GPs	Multivariate
**Chazan, B (2007)** [25]	Israel (Northern)	PC	Py, Nu, Ph, Pa	All	200 participants	Bivariate
**Briel, M (2005)** [26]	Switzerland	PC	Py	Adults	45 Py	Multivariate Bivariate
					624 Pa	
**Monette, J (2007)** [27]	Canada	PC	Py	Geriatric patients	36 Py	Multivariate
**Enriquez-Puga, A (2009)** [28]	England	PC	Py, GPs	All	28 practices	Multivariate Bivariate
**Bjerrum, L (2006)** [29]	Spain	PC	GPs	Adults	17 GPs in IG 35 GPs in CG	___
**Mcisaac, WJ (2002)**[30]	Canada	PC	GPs	Children Adults	97 Py	Multivariate Bivariate
					621 patients	
**Wheeler, JG (2001)** [31]	USA	PC	Py, Pa	Pediatric patients	16 Py	Bivariate
					771 parents	
**Juzych, NS (2005)** [32]	USA	PC	Py, Ps, Nu, Ph, Pa	Adults Children	12 Py + 9 Ps in IG	Univariate
					6 Py + 9 Ps in CG	
**Smeets, HM (2009)** [33]	Netherlands	PC	GPs, Ph, Pa	___	131 practices in IG	Multivariate Bivariate
					127 practices in CG	
**Mandryk, JA (2006)** [34]	Australia	PC	GPs	___	___	Multivariate
**Stille, CJ (2008)** [35]	USA	PC	Py, Pa	Pediatric patients	168 Py	Multivariate Bivariate
**Finkelstein, JA (2001)** [36]	USA	PC	Py, Pa	<6 years	14468 Pa (pre-)	Multivariate Bivariate
					13461 Pa (post-)	
**Altiner, A (2007)** [37]	Germany	PC	GPs, Pa	≥16 years	104 GPs (pre-)	Multivariate
					28 GPs + 787 Pa in CG	
					33 GPs + 920 Pa in IG	
**Légaré, F (2010)** [38]	Canada	PC	Py	All	18 Py in IG + 15 Py in IG	Multivariate
					245 Pa in IG + 214 Pa In CG	
**Kiang, KM (2005)** [39]	USA	PC	Py, GPs, Ps, Nu, Pa, O	Adults and pediatric patients	1800 Py	Multivariate
**Mohagheghi, MA (2005)** [40]	Iran	PC	GP	Adults	40 GPs in CG	___
					40 GPs in IG	
**Irurzun, C (2005)** [41]	Argentina	PC	Py	≥15 years	19 Py	Bivariate
**Chalker, J (2005)** [42]	Vietnam and Thailand	Pharmacy	Ph	___	124 pharmacies	Multivariate
**Finkelstein, JA (2008)** [43]	USA	PC	Py, Pa	≤6 years	223 135 person/years	Multivariate
**Chuc, NTK (2002)** [44]	Vietnam	Pharmacy	Ph	___	58 pharmacies	Bivariate
**Belongia, EA (2001)** [45]	USA	PC	Ps, Pa	Children	109 Py in IG	Multivariate Univariate
					52 in CG	
**Belongia, EA (2005)** [46]	USA	PC	Py, Ps, Pa	___	12790 Py	Multivariate Univariate
**Greene, RA (2004)** [47]	USA	PC	Py, Ps	Adults Children	900 Py and Pa	Bivariate
**Teng, CL (2007)** [48]	Malaysia	PC	GPs	___	29 GPs	Bivariate
**Awad, AI (2006)** [49]	Sudan	PC	GPs	___	1800 Pa	Bivariate
**Welschen, I (2004)** [50]	Netherlands	PC	GPs, Ph, Pa, O	___	89 GPs	Bivariate
**Gonzales, R (2004)** [51]	USA	PC	Py, Pa,	Elderly	51 office practice in CG	Multivariate
					4 office practices in IG	
**Colomina Rodríguez, J (2010)** [52]	Spain	PC	Py, Ph, Pa, O	All	___	Bivariate
**Hickman, DE (2003)** [53]	USA	PC	Py, Nu, Pa	Adults	___	Bivariate
				Children		
**Coenen, S (2004)** [54]	Belgium	PC	GPs	Adults	42 GPs in IG	Multivariate Bivariate
					43 GPs in CG	
**Perz, JF (2002)** [55]	USA	PC	Py, Ps, Pa	Pediatric patients	464200 person-years	Multivariate
**Sondergaard, J (2003)** [56]	Denmark	PC	Py	___	299 GPs	Bivariate
**Doyne, EO (2004)** [57]	USA	PC	Ps, Pa	Pediatric patients	6 practices - IG	Multivariate
					6 practices - CG	
**Bauchner, H (2006)** [58]	USA	PC	Ps	Children (3–36 months)	1368 Pa - IG	Multivariate Bivariate
					1138 Pa - CG	
**Christakis, DA (2001)** [59]	USA	PC	Ps, Nu, O	Children	16 providers - IG	Bivariate
					12 providers - CG	
**Smabrekke, L (2002)** [60]	Norway	PC	Ps, Nu, Pa	Children (1–5 years)	819 Pa	Bivariate
**Bjerrum, L (2011)** [61]	Several	PC	GP	Adults	47011	___
**Regev-Yochay, G (2011)** [62]	Israel	PC	GP	Children	3636	Multivariate
**Llor, C (2011)** [63]	Spain	PC	GP	___	235 (full)	Univariate Multivariate
					97 (partial)	
**Weiss, K (2011)** [64]	Canada	PC	GP	___	All GP	Multivariate
**Llor, C (2011)** [65]	Spain	PC	GP	Adults (14-60 years)	10 first patients	___
**McKay, RM (2011)** [66]	Canada	PC	Py, Ph, O	___	___	Bivariate
**Deuster, S (2010)** [3]	Switzerland	HC	Py	Adults	292 Pa	Bivariate
**Chang, MT (2006)** [67]	Taiwan	HC	GPs	___	5046 Pa (pre-)	Bivariate
					5054 Pa (post-)	
**Naughton, BJ (2001)** [68]	USA	HC	Py, Nu	Geriatric patients	350 episodes	Bivariate
**Lutters, M (2004)** [69]	Switzerland	HC	Py	Geriatric patients	3383 Pa	Bivariate
**Loeb, M (2005)** [70]	Canada and USA	HC	Py, Nu	Geriatric patients	4217 residents	Bivariate
**Lesprit, P (2009)** [71]	France	HC	Py	___	786 Pa	Bivariate
**Akter, SFU (2009)** [72]	Bangladesh	HC	Py	Pediatric patients	2171 Pa (pre-)	Bivariate
					1295 Pa (post-)	
**Paul, M (2006)** [73]	Israel	HC	Py	Adults	1203 Pa (pre-)	Bivariate
	Germany					
	Italy				2326 Pa (post-) (1245 IG and 1801 CG)	
**Camins, BC (2009)** [74]	USA	HC	Py (internists)	___	784 new prescriptions	Multivariate Bivariate
**Westphal, JF (2010)** [75]	France	HC	Py	___	471 cases of pneumonia 104 (pre-); 367 (post-)	Bivariate
**Mullet, CJ (2001)** [76]	USA	HC	Ps, Nu	Pediatric > 6 months	809 Pa (pre-)	Bivariate
					949 Pa (post-)	
**von Gunten, V (2005)** [77]	Switzerland	HC	Py	___	1200 Pa	Multivariate Bivariate
**Ansari, F (2003)** [78]	UK	HC	Py	___	40 medical and surgical wards	Multivariate
**Kisuule, F (2008)** [79]	USA	HC	Py, Nu	___	17 hosp. practitioners	Bivariate
**Halm, EA (2004)** [80]	USA	HC	Py, Nu, Pa, O	Adults	2094 cases	Bivariate
					1013 (pre-)	
					1081 (post-)	
**López-Medrano, F (2005)** [81]	Spain	HC	Py	___	1280 treatments	Bivariate
**Agwu, AL (2008)** [82]	USA	HC	Py, Ps	Children	___	Bivariate
**Barenfanger, J (2001)** [83]	USA	HC Pharmacy	Ph	___	378 Pa (188 IG and 190 CG)	Bivariate
**Rϋttiman, S (2004)** [84]	Switzerland	HC	Py	Adults	500 Pa	Bivariate
**Martin, C (2005)** [85]	USA	HC	Py	___	___	____
**Solomon, DH (2001)** [86]	USA	HC	Py	___	4500 Pa	Bivariate
**Fowler, S (2007)** [87]	UK	HC	Py	Elderly ≥ 80 years	6129 admissions	Multivariate
**Sintchenko, V (2005)** [88]	Australia	HC	Py	___	12 internists	Bivariate
**Yong, MK (2010)** [89]	Australia	HC	Py	___	___	Bivariate
**Meyer, E (2010)** [90]	Germany	HC	Py	Adults	4684 Pa (pre-)	Multivariate
					7203 Pa (post-)	
**Thursky, KA (2006)** [91]	Australia	HC	Py	Adults	489 Pa (pre-)	Multivariate
					497 Pa (post-)	
**Petterson, E (2011)** [92]	Sweeden	HC	Nu, Py	Elderly	60 residents	___
**Tangden, T (2011)** [93]	Sweeden	HC	Py	elderly	___	Multivariate
						Bivariate
**Talpaert, MJ (2011)** [94]	UK	HC	Py	Adults	___	Multivariate
**Bevilacqua, S (2011)** [95]	France	HC	Py	Adults	___	Bivariate
**Shen, J (2011)** [96]	China	HC	Py	Adults	354 patients	Multivariate
						Bivariate

(a) PC – primary care; HC – hospital care.

(b) GPs – general practitioners; Ps – pediatrics; Py – physicians; Pa – patients or their caregivers; Ph – pharmacists; Nu – nurses; O – others.

(c) CG – control group; IG – intervention group.

In primary care (table 2), 33 studies (70%) [20–24, 26, 27, 29, 30, 32–39, 41, 44, 45, 47, 48, 50, 51, 53, 54, 56, 58–61, 63, 65] focused on the use of antibiotics in respiratory infections, and one focused on the use of antibiotics in infectious diseases and other infections (urinary infections, skin and soft tissue infections and septicemia) [27]; the remaining 30% failed to identify any target disease [25, 28, 31, 40, 42, 43, 46, 49, 52, 55, 57, 62, 64, 66]. Of the 47 papers, 27 (57%) studied the efficacy/effectiveness of one or more interventions versus non-intervention, using a control group that received the intervention in four studies, dissemination of guideline information in three [56, 57, 65] and educational sessions on diagnosis of *otitis media* in one [58]. In this last study, the educational session in the intervention group included diagnosis of *otitis media* and information on recommendations for antibiotic use.

**Table 2:** **Interventions to improve antibiotic use in primary care**

Author (year)	Study design (a)	Program description		Baseline and follow-up			Analysis (e)	Results (f)
		Disease (b)	Intervention type (c,d)	Baseline	Intervention period	Follow-up		
**Dollman, WB (2005)** [20]	1	URTI	IG: 1, 2, 8	5 months	5 months	___	2	T (+)
			CG: 0					
**Hrisos, S (2008)** [21]	4	URTI	IG1: 3	___	3 months	___	3	At/Bh (+)
			IG2: 3					
			IG1 + 2: 3					
			CG: 0					
**Hennessy, TW (2002)** [22]	3	RTI	IG: 8, 2	2 months	12 months (6 each year of intervention)	2 months	2, 3	T (+)
			CG: 0					
**Rubin, MA (2005)** [23]	2	URTI	IG: 1, 2, 8, 9	6 months	6 months	___	2, 3	T (+)
			CG: 0					Ga (+)
**Naughton, C (2009)** [24]	4	RTI	IG1: 3, 4	12 months	___	12 months	2, 3	T (+) (−)^a^
			IG2: 3					Ga (+) (−)^a^
**Chazan, B (2007)** [25]	1	Infectious disease	IG1: 1, 2	4 months	4 months	___	2, 3	T (+)
			IG2: 1, 2, 8					
**Briel, M (2005)** [26]	4	ARTI	IG1: 1,2	___	5 months	___	1	T (+)
			IG2: 1,2					
			CG: 0					
**Monette, J (2007)** [27]	4	Lower RTI	IG: 1, 3	3 months	2 x 3 months	3 months	5, 6	Ga (+)
		UTI	CG: 0					
		Skin and soft-tissue infections septicemia						
**Enriquez-Puga, A (2009)** [28]	4	___	IG: 1, 3, 4	2 periods of 6 months	6 months	24 months	5, 6	Ga (−)
**Bjerrum, L (2006)** [29]	2	RTI	IG: 2, 3, 10	3 weeks during 3 months	3 weeks during 3 months	___	1, 2	T (+)
			CG: 0					Ga (+)
**Mcisaac, WJ (2002)** [30]	4	Sore throat	IG: 1, 5	___	___	___	1	T (−)
			CG: 0					Ga (−)
**Wheeler, JG (2001)** [31]	1	Viral infections	IG: 2, 8, 9	1 week	3 weeks during 3 years	6 months (qualitative)	3	T (−)
								At/Bh (+)
**Juzych, NS (2005)** [32]	3	URTI	IG: 1, 2, 8	4.5 months	4.5 months	___	2, 3	Pa (+) (−)^b^
			CG: 0					T (+)
**Smeets, HM (2009)** [33]	2	RTI	IG: 2, 3, 8	6 months	6 months	6 months (one year later)	5, 6	T (−)
								Ga (−)
			CG: 0					
**Mandryk, JA (2006)** [34]	1	URTI	IG: 1, 2, 3, 4	33 months	51 months	___	2	Ga (+)
								T (+)
**Stille, CJ (2008)** [35]	4	RTI	IG: 1, 2, 8	---	---	6 months	1	At/Bh (+) (−)^c^
			CG: 0					
**Finkelstein, JA (2001)** [36]	4	*Otitis media*	IG: 1, 2, 3, 8	12 months	12 months	___	2, 3	T (+)
		Pharyngitis	CG: 0					
		Sinusitis						
		Cold						
		Bronchitis						
**Altiner, A (2007)** [37]	4	Acute cough	IG: 4, 8	3 months	___	3 months after 6 weeks 3 months after 1 year after	5, 6	T (+)
			CG: 0					
**Légaré, F (2010)** [38]	4	Acute RI	IG: 1, 2	___	___	___	2, 3	T (+)
			CG: 0					
**Kiang, KM (2005)** [39]	1	Respiratory illnesses	IG: 1, 2, 8	___	___	___	2, 3	Ga (+)
								At/Bh (+)
**Mohagheghi, MA** [40]	4	___	IG: 2	60 months	___	3 months afterwards 1 year afterwards	2, 3	T (+) (−)^d^
			CG: 0					
**Irurzun, C (2005)** [41]	1	Pharyngitis and tonsillitis	IG: 1, 2, 3, 4, 10	___	12 months	___	2	T (+)
								Ga (+)
**Chalker, J (2005)** [42]	5	___	IG: 2, 4, 11	___	___	3x3 months (one month after each intervention)	1	T (+) (−)^e^
			CG: 0					
**Finkelstein, JA (2008)** [43]	4	___	IG: 1, 2, 3, 8	24 months	6 months during 3 years	___	2, 3	T (+) (−)^f^
			CG: 0					Ga (+)
**Chuc, NTK (2002)** [44]	4	ARTI	IG: 2, 4, 11	___	___	___	2, 3	T (+)
								Qh (+)
**Belongia, EA (2001)** [45]	3	ARTI	IG: 1, 2, 8	6 months	___	6 months (every two years)	7, 8	T (+)
**Belongia, EA (2005)** [46]	2	___	IG: 1, 2, 8, 9	12 months	48 months	___	3, 4	T (+) (−)^g^
**Greene, RA (2004)** [47]	1	Acute sinusitis	IG: 1, 2, 3, 13	22 months	14 months	___	2	Ga (+)
								T (+)
**Teng, CL (2007)** [48]	2	URTI and others	IG: 1, 2, 4	3 months	___	3 months	2	T (+)
**Awad, AI (2006)** [49]	4	___	CG: 0	___	___	1 and 3 months afterwards	2, 3	T (+)^h^
			IG1: 1, 3					Ga (+)
			IG2: 2, 3					
			IG3: 3, 4					
**Welschen, I (2004)** [50]	4	ARTI	IG: 1, 2, 3, 8	3 months	___	3 months	2, 3	T (+)
**Gonzales, R (2004)** [51]	2	ARTI	IG: 1, 8	4 months	4 months (study period)		2, 3	T (+) (−)^i^
**Colomina Rodríguez, J (2010)** [52]	1	___	IG: 1, 2, 6, 8	48 months	36 months	24 months	5	T (+)
								Ga (+)
**Hickman, DE (2003)** [53]	4	Acute bronchitis	IG: 1, 2, 8	6 months	___	6 months	2, 3	T (+)
			CG: 0					
**Coenen, S (2004)** [54]	4	Acute cough	IG: 1, 4	3 months	1 month (without outcomes)	___	2,3	T (+)
			IG: 0					Ga (+) (−)^j^
**Perz, JF (2002)** [55]	1	___	IG: 1, 2, 8, 9	12 months	12 months	12 months	5,6	T (+)
			CG: 0					
**Sondergaard, J (2003)** [56]	4	RTI	IG: 1, 3	3 periods of 3 months	3 periods of 3 months	3 months (not shown)	2,3	T (−)
			CG: 1					Ga (−)
**Doyne, EO (2004)** [57]	4	___	IG: 1, 2, 3, 8	12 months	12 months	___	2,3	T (+) (−)^k^
			CG1: 1, 3					
			CG: 1					
**Bauchner, H (2006)** [58]	5	Acute *otitis media*	IG: 1, 2, 3	___	___	___	1	Ga (+) (−)^L^
			CG: 2					
**Christakis, DA (2001)** [59]	4	Acute *otitis media*	IG: 6	7 months	8 months	___	2,3	T (−)
			CG: 0					Ga (+)
**Småbrekke, L (2002)** [60]	2	Acute *otitis media*	IG: 1, 2, 8	4 months	4 months	___	2,3	T (+)
			CG: 0					Ga (+)
**Bjerrum, L (2011)** [61]	1	RTI	IG = 2, 3, 9, 10	3 weeks (x2years)	3 weeks (x1 year)	___	2, 3	T (+)
								Ga (+)
**Regev-Yochay, G (2011)** [62]	4	___	IG = 2	2 years	1 year	___	2, 3	T (+)
			CG = 0					Ga (+)
**Llor, C (2011)** [63]	4	Pharyngitis	IG1 = 2, 8, 10	15 days	15 days	___	2, 3	T (+)
			IG2 = 2, 8, 10 (sem)					
**Weiss, K (2011)** [64]	1	___	IG = 1	2 years	7 years	___	2, 3	T (+)
			CG = 0					
**Llor, C (2011)** [65]	4	Acute pharyngitis	IG = 1, 10	___	___	___	1	Ga (+)
			CG = 1					
**McKay, RM (2011)** [66]	1	___	IG = 1, 2, 8, 9	9 years	3 years	___	2	Pa (+) (−)^m^

^*a*^*In*[24]*, significantly positive in post-intervention period but no significant change post-follow-up.*

^*b*^*In*[32]*, while prescriptions for pharyngitis, otitis media and URTI decreased significantly post-intervention, the decrease in the case of bronchitis was not as significant.*

^*c*^*In*[35]*, comparison between attitudes, knowledge and behavior of physicians in the intervention versus the control group showed no significant differences. Physicians in the intervention group reported that they had changed their prescribing in the preceding 3 years.*

^*d*^*In*[40]*, after one year, there was a reduction in the percentage of antibiotic prescribing in the intervention group but this was not statistically different from the control group.*

^*e*^*In*[42]*, interventions resulted in improved antibiotic use, which was statistically significant in the Hanoi but not in the Bangkok study.*

^*f*^*In*[43]*, there was no significant decrease in one age group (3–24 months).*

^*g*^*In*[56]*, the reduction in antibiotic prescribing by pediatricians was greater in the control than in the intervention group.*

^*h*^*In*[49]*, audit and feedback combined with academic detailing or seminars appeared to be more effective in changing antibiotic prescribing practices than audit and feedback alone.*

^*i*^*In*[51]*, there was a moderate decrease in total antibiotics prescribed but this was not statistically significant.*

^*j*^*In*[54]*, appropriate antibiotic prescribing improved post-intervention but did not prove statistically significant.*

^*k*^*In*[57]*, the prescribing rate decreased in all groups but there were no statistically significant differences between groups.*

^*L*^*In*[58]*, adherence was high though not statistically significant in the intervention group, but, in second episodes there were no differences in adherence, between groups.*

^*m*^*In*[66]*, utilization rates for acute bronchitis are at the same level as when intervention began, but other acute respiratory tract infections declined.*

**(a) Disease:** URTI – upper respiratory tract infections; RTI – respiratory tract infections; ARTI – acute respiratory tract infections; UTI – urinary tract infections.

**(b) Study design (SD):** (1) before/after studies; (2) – nonrandomized controlled trial without cross-contamination control; (3) – nonrandomized controlled trial with cross-contamination control; (4) - randomized controlled trial without cross-contamination control; (5) - randomized controlled trial with cross-contamination control.

**(c)** IG – intervention group; CG – control group.

**(d) Type of intervention (TI):** (0) no intervention; (1) dissemination of printed/audiovisual educational materials (mailed printed matter; protocols and guidelines; self-instruction materials; drug bulletins); (2) group education, including group-session rounds, conferences, lectures, seminars and tutorials; (3) feedback of physician prescribing patterns (individually or including a comparison of these patterns with peer behavior and/or accepted standards) or feedback of patient-specific lists of prescribed medication; (4) individual outreach visits; (5) reminders at the time of prescribing; (6) computer-assisted decision-making systems; (7) formulary-control/restrictive formulary process; (8) patient education (pamphlets); (9) patient education (videotapes); (10) workshops on rapid tests / introduction of Rapid Antigen Detection Tests (RADTs) in consulting offices; (11) enforcement of regulations; (12) prescription feedback with recommendations to modify it by pharmacists and/or infectious-disease physicians; (13) financial incentives.

**(e) Type of data-analysis (T):** (1) comparison of post-test values between groups; (2) comparison of pre- and post-values within each group; (3) comparison of pre- and post-values between groups; (4) comparison of follow-up values between groups; (5) comparison of pre-, post- and follow-up values within each group; (6) comparison of pre-, post- and follow-up values between groups.

**(f) Results analyzed (R):** (T) total antibiotics prescribed/dispensed; (Ga) choice of appropriate antibiotics/adherence to antibiotic guidance according to guideline algorithms, including dosages and routes of administration; (Pa) prescription rate per disease; (At/Bh) attitudes and behavior; (Qph) quality of pharmacy practice.

Only two studies [51, 64], evaluated the efficacy of passive interventions in physicians and in one of them interventions was in combination with educational campaigns directed at patients and their caregivers [51]. All the other studies included active interventions in health professionals (whether or not associated with passive interventions). Three studies [22, 46, 66], involved active interventions in patients and health professionals, and in four studies [26, 33, 50, 62] the interventions included improvement of doctor-patient communication skills.

Twenty-nine studies (62%) [20–23, 25–27, 29, 34, 36–39, 41, 44, 45, 47–50, 52, 53, 55, 60–65] reported positive results for all outcomes measured; fourteen studies (30%) [24, 31, 32, 35, 40, 42, 43, 46, 51, 54, 57–59, 66] reported positive results for some outcomes, and results that were not statistically influenced by the intervention for others; only four studies [28, 30, 33, 56] failed to report significant post-intervention improvements for all outcomes.

While some studies conducted no post-intervention follow-up of participants [20, 21, 23, 25, 26, 29, 30, 32, 34, 36, 38, 39, 41, 43, 44, 46, 47, 54, 57–66], others followed up their participants for different periods, ranging from two months [22] to three [27, 37, 40, 42, 48–50, 56], six [31, 33, 35, 45, 53], twelve [24, 55] and twenty-four months [28, 52].

Interventions that included improving diagnostic procedures to help physicians distinguish bacterial from viral infections led to very positive results [29, 41, 61, 63, 65].

### Interventions in hospital care professionals

Whereas most interventions concentrated on physicians (Table 1), some included a multidisciplinary intervention targeting physicians and nurses [68, 70, 76, 79, 92], patients [80], and in one case, solely pharmacists [83]. Some studies identified the patients targeted, with these being elderly in five instances [68–70, 87, 92, 93] children in three [72, 76, 82]. Table 3 summarizes the studies retrieved containing interventions for improving antibiotic use in hospital care. The diseases targeted were as follows: pneumonia in four cases [68, 75, 80, 93]; urinary infections in two [70, 72]; urinary and upper respiratory tract infections in one [69]; pneumonia, meningitis and urinary infection in one study [85], and bronchitis, community acquired pneumonia and chronic obstructive pulmonary disease in other [96]. Of the thirty-one papers, 6 (20%) studied the efficacy/effectiveness of one or more interventions versus no intervention, using a control group [70, 71, 73, 77, 83, 86]. Naughton [68] compared two strategies, a multidisciplinary intervention in physicians and nurses, and a physician-only intervention in ten skilled nursing facilities randomized into two groups, and reported no statistically significant differences between the two groups. Most of the reported hospital-based interventions coincided with the implementation of protocols or new computer systems, with the result that post-intervention were compared with pre-intervention outcomes without the use of control groups.

**Table 3:** **Interventions to improve antibiotic use in hospital settings**

*Author (year)*	*Study design (a)*	*Program description*		*Baseline and follow-up*			*Analysis (d)*	*Results (e)*
		Disease	Intervention type (b, c)	Baseline	Intervention period	Follow-up		
**Deuster, S (2010)** [3]	1	Most common hospital infections	IG: 1, 2	8 weeks	8 weeks	8 weeks (1 year after)	5	Ga (+) (−)^a^
**Chang, MT (2006)** [67]	1	___	IG: 1, 7	3 months	3 months	___	2	T (+)
								Ga (+)
**Naughton, BJ (2001)** [68]	4	Pneumonia	IG: 1, 2	6 months	6 months	___	2, 3	T (−)
			CG: 1, 2					
**Lutters, M (2004)** [69]	1	RTI and UTI	IG: 1, 2, 4	12 months	24 months	___	2	T (+)
								Ga (+)
**Loeb, M (2005)** [70]	4	UTI	IG: 1, 2, 4	___	___	___	1	T (+)
			CG: 0					
**Lesprit, P (2009)** [71]	2	Various	IG: 1, 2, 12	___	8 weeks	___	1	Ga (+)
			CG: 1, 2					
**Akter, SFU (2009)** [72]	2	Common pediatric infections	IG: 2	4 months	4 months	___	2, 3	T (+)
								Ga (+)
**Paul, M (2006)** [73]	5	___	IG: 6	7 months	7 months	___	1, 2	Ga (+)
			CG: 0					
**Camins, BC (2009)** [74]	4	___	IG: 1, 3, 4	___	10 months	___	1	Ga (+)
			CG: 1 (guidelines)					
**Westphal, JF (2010)** [75]	1	Pneumonia	IG: 2, 5, 6	18 months	54 months	___	2	Ga (+) (−)^b^
**Mullet, CJ (2001)** [76]	1	___	IG: 6	6 months	6 months	___	2	T (+) (−)^c^
								Ga (+)
**von Gunten, V (2005)** [77]	5	___	IG_B_: 1	6 months	6 months	___	2, 3	T (+)
			IG_C_: 1, 2, 12					Ga (+)
			CG_A_: 0					
**Ansari, F (2003)** [78]	1	___	IG: 12	24 months	24 months	___	2	Ga (+) (−)^d^
								T (+)
**Kisuule, F (2008)** [79]	1	___	IG: 1, 3, 4	Period until 20 prescriptions	2 months	1 month	2	Ga (+)
**Halm, EA (2004)** [80]	1	Pneumonia	IG: 1, 2, 8, 9	5 months	---	5 months	2	Ga (+)
**López-Medrano, F (2005)** [81]	1	___	IG: 12	12 months	12 months	___	2	T (+)
								Ga (+)
**Agwu, AL (2008)** [82]	1	___	IG: 6, 12	12 months	12 months	___	2	Ga (+)
**Barenfanger, J (2001)** [83]	4	___	IG: 6	___	5 months	___	1	T (+)
			CG: 0					
**Rüttiman, S (2004)** [84]	1	___	IG: 1, 2, 3	___	___	___	2	T (+) Ga (+)
**Martin, C (2005)** [85]	1	Pneumonia	IG: 1, 2	___	60 months	___	2	Ga (+)
		Meningitis						
		UTI						
**Solomon, DH (2001)** [86]	4	___	IG: 1, 3, 4, 12	4 weeks	18 weeks	___	2, 3	Ga (+)
			CG: 0					
**Fowler, S (2007)** [87]	1	___	IG: 1, 3	21 months	21 months	___	2	Ga (+)
**Sintchenko, V (2005)** [88]	1	Intensive care	IG: 6	6 months	6 months	___	2	T (+) Ga (+)
**Yong, MK (2010)** [89]	1	Intensive care	IG: 6	30 months	54 months	___	2	Ga (+)
**Meyer, E (2010)** [90]	1	Intensive care	IG: 2	24 months	36 months	___	2	T (+)
**Thursky, KA (2006)** [91]	1	Intensive care	IG: 2, 6	6 months	6 months	___	2	T (+) Ga (+)
**Petterson, E (2011)** [92]	4	UTI	IG = 1, 2, 3	3 months	3 months		2, 3	T (+)
			CG = 0					Ga (+)
**Tangden, T (2011)** [93]	1	Pneumonia (Intravenous)	IG = 1, 2	7 years		2.5 years	3	T (+)
								Ga (+) (−)^e^
**Talpaert, MJ (2011)** [94]	1	___	IG = 2	12 months	12 months		3	T (+) (−)^f^
**Bevilacqua, S (2011)** [95]	2	___	IG = 3, 7, 12	12 months	12 months		2, 3	Ga (+)
			CG = 0					
**Shen, J (2011)** [96]	2	Bronchitis	IG = 12		10 months		1	Ga (+)
		Community acquired pneumonia	CG = 0					
		Acute exacerbation of COPD						

^*a*^*In*[3]*, the follow-up analysis showed sustained adherence to guidelines in hospital-acquired pneumonia but a decrease in guideline adherence in the case of UTI.*

^*b*^*In*[75]*, there was a significant decrease in the proportion of antibiotic orders containing at least one criterion that was not in line with the guideline, but the choice of antibiotics according to the context of acquisition of pneumonia, improvement was not statistically significant.*

^*c*^*In*[76]*, total of antibiotics used was similar but the number of orders placed per antibiotic course decreased post-intervention.*

^*d*^*In*[78]*, there was a significant decrease in use of total and alert antibiotics, except in the case of ceftriaxone and mercapen.*

^*e*^*In*[93]*, there was a reduction of cefalosporines consumption, but pipiracillin/tazobactan and penicillin increased*

^*f*^*In*[94]*, there was a reduction in fluorquinolone and cefalosporine but no significant change total of antibiotics neither clindamicine, amoxiciline and co-amoxclav use.*

**(a) Disease:** URTI – upper respiratory tract infections; RTI – respiratory tract infections; ARTI – acute respiratory tract infections; UTI – urinary tract infections; COPD-Chronic obstructive pulmonary disease.

**(b) Study design (SD):** (1) before/after studies; (2) – nonrandomized controlled trial without cross-contamination control; (3) – nonrandomized controlled trial with cross-contamination control; (4) - randomized controlled trial without cross-contamination control; (5) - randomized controlled trial with cross-contamination control.

**(c)** IG – intervention group; CG – control group.

**(d) Type of intervention (TI):** (0) no intervention; (1) dissemination of printed/audiovisual educational materials (mailed printed matter; protocols and guidelines; self-instruction materials; drug bulletins); (2) group education, including group-session rounds, conferences, lectures, seminars and tutorials; (3) feedback of physician prescribing patterns (individually or including a comparison of these patterns with peer behavior and/or accepted standards) or feedback of patient-specific lists of prescribed medication; (4) individual outreach visits; (5) reminders at the time of prescribing; (6) computer-assisted decision-making systems; (7) formulary-control/restrictive formulary process; (8) patient education (pamphlets); (9) patient education (videotapes); (10) workshops on rapid tests / introduction of Rapid Antigen Detection Tests (RADTs) in consulting offices; (11) enforcement of regulations; (12) prescription feedback with recommendations to modify it by pharmacists and/or infectious-disease physicians; (13) financial incentives.

**(e) Type of data-analysis (T):** (1) comparison of post-test values between groups; (2) comparison of pre- and post-values within each group; (3) comparison of pre- and post-values between groups; (4) comparison of follow-up values between groups; (5) comparison of pre-, post- and follow-up values within each group; (6) comparison of pre-, post- and follow-up values between groups.

**(f) Results analyzed (R):** (T) total antibiotics prescribed/dispensed; (Ga) choice of appropriate antibiotics/adherence to antibiotic guidance according to guideline algorithms, including dosages and routes of administration; (Pa) prescription rate per pathology: (At/Bh) attitudes and behavior; (Qph) quality of pharmacy practice.

While some studies [67, 73, 76, 83, 87–89] used passive interventions, all the others used active interventions or passive and active simultaneously. Twenty-four papers (78%) [67, 69–74, 77, 79–92, 95, 96] reported positive results for all outcome measures; 6 papers (20%) [3, 75, 76, 78, 93, 94] reported some outcomes as positive and others as positive statistically non-significant; and Naughton reported negative results [68].

In contrast to primary care in which only three studies [24, 26, 32] analyzed clinical outcomes, in hospital care some studies [67, 69, 70, 72, 74, 81, 84, 86, 96] compared outcomes pre- and post-intervention to assess whether a reduction in antibiotic use might cause clinical alterations, and no influences were observed, namely, to length of hospital stay, and mortality, morbidity and/or readmission rates.

Many of the hospital-care studies highlighted the important role of clinical pharmacists in drawing up and implementing guidelines and policies for antibiotic use in hospital settings [3, 67, 69, 74, 75, 77–80, 82, 85, 86, 91, 96].

### Studies design

While 25 papers (53%) [21, 24, 26–28, 30, 35–38, 40, 42–44, 49, 50, 53, 54, 56–59, 62, 63, 65] reported randomized controlled studies in the case of primary care, a far lower number, i.e., 8 (26%) [68, 70, 73, 74, 77, 83, 86, 92], reported this type of study in the case of hospital care, and only one of these included cross-contamination control. Cross-contamination can occur when the participants of different intervention or control groups have close working relationships and might share information about the intervention, and this is important because differences in the results between the intervention and the control group may be influenced by this factor. In some studies physicians participated on a voluntary basis (they were invited to participate in the study), and their prescribing habits recorded during the intervention may not represent their real use of antibiotics [24, 26–30, 33, 37, 50, 61–63, 65, 70].

There were many differences in the analytical approaches adopted by the different studies: while some compared the results of the intervention with the situation at baseline, and some compared the results between groups pre- and post-intervention, others focused exclusively on the position post-intervention. There were few studies that conducted a follow-up after the intervention had ended, and those which did reported that the majority of positive results observed in the post-intervention period were lost over time.

No studies were found in which the interventions had been designed on the basis of the attitudes and behavior responsible for antibiotic prescribing or dispensing habits, despite the fact that many authors contend that this knowledge contributes to the success of educational interventions in health professionals [69, 79, 80]. In some studies [28, 33, 49, 79], however, interventions addressed barriers facing the individual prescriber, particularly when it came to dealing with diagnostic uncertainty, and were tailored to: overcoming any identified barriers and enable general practitioners (GPs) to reflect on their own prescribing; helping decrease uncertainty about appropriate disease management and appropriate prescribing; facilitating more patient-centered care; and being beneficial to implementation in practice. One study [39] assessed the impact of interventions on the knowledge, beliefs, and decision-making of primary care physicians, and two others, used workshops and focus-group discussions to determine the possible motivating factors underlying observed prescribing practices [49, 62]. The importance of interventions being acceptable to physicians was highlighted by a recent systematic review [97].

All the studies underlined the importance of appropriate use of antibiotics to prevent the problem of microbial resistance, and stated that the most important aim of interventions to improve antibiotic use was to reduce this important public health problem. Even so, only one primary-care [46] and eight hospital-care studies [67, 84, 85, 87, 89–91, 93] analyzed improvement in bacterial susceptibility during the intervention. While some studies reported the reduction in the cost of antibiotic use, only five studies analyzed the effectiveness of intervention in terms of the cost of the intervention versus the cost of reducing antibiotic use [73, 78, 81, 84, 86, 96].

We found only two studies that addressed interventions (undertaken in Thailand and Vietnam, respectively) [42, 44] specifically designed to improve pharmacists’ to combat the dispensing of antibiotics without prescription, despite there were studies which established that the sale of antibiotics without a prescription are a reality in some European countries [98–102]. Although some of the studies reviewed -mainly those pertaining to hospital care- reported the important role played by pharmacists in developing interventions to be undertaken in physicians and implementing antibiotic treatment guidelines and protocols in hospital settings, there were few studies with interventions targeted at pharmacists. Some authors stressed the usefulness of including pharmacists in teams tasked with drawing up recommendations and making decisions about antibiotic use in certain countries [54, 96, 103, 104].

Results obtained by our search showed that the majority of published studies about educational interventions describe active and multifaceted interventions. This finding is in accordance with a number of systematic meta-analyses of randomized controlled trials to improve health care practice, which conclude that highly interactive learning methods, such as educational outreach visits [105] workshops [106, 107], small discussion groups [107, 108], individualized training sessions [107, 108], practice-based interventions [19] and case-based learning [109], are the most effective strategies.

Some recent review papers on interventions to improve antibiotic prescribing [9–11, 97] (Table 4) focus on a limited set of intervention targets, such as acute outpatient infections, and more specifically on clinical knowledge and decision-making processes [9], specific populations (children), specific diseases (upper respiratory tract infections) [10] or purpose-designed noneducational (stewardship) interventions in specific hospital divisions, such as critical care [11] and acute care [12]. One paper [97] reviewed studies that evaluated GPs’ perceptions about antibiotic prescribing and interventions aimed at prudent prescribing. Our study only analyzed educational interventions but was more extensive, in that it included interventions aimed at physicians and/or pharmacists in both primary-care and hospital settings, and focused on any disease with antibiotic prescribing for child, adult or geriatric patients. In contrast to Steinman [9], who made a quantitative analyses of quality-improvement strategies, our review, like those of Boonacker [10], Kaki [11] and Charani [12], was a qualitative analysis.

**Table 4:** **Review studies covering interventions to improve antibiotic use**

Author (year)	Title of study	Study objectives	Inclusion criteria	Methods	Number of studies included	Review period
van der Velden (2012) [13]	Effectiveness of physician-targeted interventions to improve antibiotic use for respiratory tract infections	To assess the effectiveness of physician-targeted interventions aiming to improve antibiotic prescribing for respiratory tract infections in primary care, and to identify intervention features mostly contributing to intervention success.	Studies with an intervention primarily targeted at physicians in a primary care setting aiming to improve antibiotic prescribing for RTIs, conducted in a high-income country, presenting a standardized outcome of (first choice) prescription measured in defined daily dosage, prescription or rates.	Systematic review of studies published in MEDLINE, EMBASE, and the Cochrane Library. Quantitative analysis to assess the association between effectiveness rates and intervention features.	58	January 1990 through July 2009
Charani, E (2011) [12]	Behaviour Change Strategies to Influence Antimicrobial Prescribing in Acute Care: A Systematic Review	To assess the effectiveness of antimicrobial prescribing interventions that either alone or in combination, aim to influence behaviors in acute care.	Effective Practice and Organization of Care (EPOC) model was adapted to include additional criteria for review of uncontrolled studies. Studies were included only if they were conducted in countries defined as having a developed health care system.	Systematic review of studies published in MEDLINE, Applied Social Sciences Index and Abstracts (ASSIA), Business Source Complete, The Cochrane Library, PsycINFO, and the Database of Abstracts of Reviews of Effectiveness (DARE) and Health Management Information Consortium (HMIC)	10	January 1999 through April 2011
Tonkin-Crine, S (2011) [97]	Antibiotic prescribing for acute respiratory tract infections in primary care: a systematic review and meta-ethnography.	To evaluate general practitioners’ perceptions about antibiotic prescribing, and interventions aimed at prudent prescribing.	Studies that used qualitative methods and analysis.	Meta-synthesis of qualitative research examining GP attitudes and experiences of antibiotic prescribing, and interventions aimed at more prudent prescribing for ARTI.	12	1950-May 2011
Kaki, R (2011) [11]	Impact of antimicrobial stewardship in critical care: a systematic review.	To evaluate the evidence for antimicrobial stewardship interventions in the critical care unit.	Studies that evaluate the effectiveness of application of any intervention to improve antimicrobial utilization and within an intensive care setting, using a modified Cochrane Registry EPOC Database inclusion criteria.	Systematic review of studies published in OVID MEDLINE, Embase and Cochrane databases	24	January 1996 through December 2010
Boonacker, CWB (2010) [10]	Interventions in health care professionals to improve treatment in children with upper respiratory tract infections.	To analyze which strategies are used to promote evidence-based interventions in the management of children with URTI and assess the related effectiveness and costs.	Randomized controlled trials, non-randomized controlled trials and controlled before/after studies using implementation methods to change health care professionals’ attitudes to the treatment of children with URTI and investigate the effectiveness of implementation strategies.	Systematic review of studies published in Pubmed, Embase and Cochrane Central Register of Controlled Trials.	17	Last search, February 2009
Steinman, MA (2006) [9]	Improving antibiotic selection. A systematic review and quantitative analysis of quality improvement strategies.	To assess which interventions are most effective in improving the prescribing of recommended antibiotics for acute outpatient infections.	Clinical trials with contemporaneous or strict historical controls that reported data on antibiotic selection in acute outpatient infections	Systematic review with quantitative analysis of the EPOC Database, supplemented by MEDLINE and hand-searches	24	Last search, November 2004

As in the case of any systematic review, ours suffers from the limitation of publication bias. The inclusion criteria allowed for the review to cover a wide range of studies with different designs, something hindered us in making comparisons and performing a meta-analysis. Identification of the design proved a complex task, and it is therefore possible that some study may have been misclassified as regards design, due to an incomplete description of the methodology used. In many cases, deficiencies in the design and description of the intervention and identification of the sample made tabulating the study characteristics difficult.

## Conclusions

The results yielded by our search show that there are many more papers on educational interventions in physicians than pharmacists. Respiratory disorders were the disease targeted by most studies, mainly in primary care. Published studies varied widely in terms of study design, outcome measures, outcome period, and definition of sample. Most studies used active or a mix of active and passive interventions, and reported that active interventions were more effective. Notwithstanding these heterogeneity, it can be concluded from the above: first, that educational interventions to improve antibiotic use are essential; and second, that in many studies such interventions are active and multifaceted, some of them include both physicians and pharmacists, and were designed taking these health professionals’ attitudes and knowledge into account, in order to focus on the barriers so identified.

## Authors’ original submitted files for images

Below are the links to the authors’ original submitted files for images. Authors’ original file for figure 1
