# Supplementary material for: Educational interventions to improve prescription and dispensing of antibiotics: a systematic review
Source: BMC Public Health. 2014 Dec 15;14:1276. doi: 10.1186/1471-2458-14-1276 (PMC4302109; doi:10.1186/1471-2458-14-1276)
Supplement: Supplementary file 1 — Authors’ original file for figure 1 [file 12889_2014_7383_MOESM1_ESM.pdf]

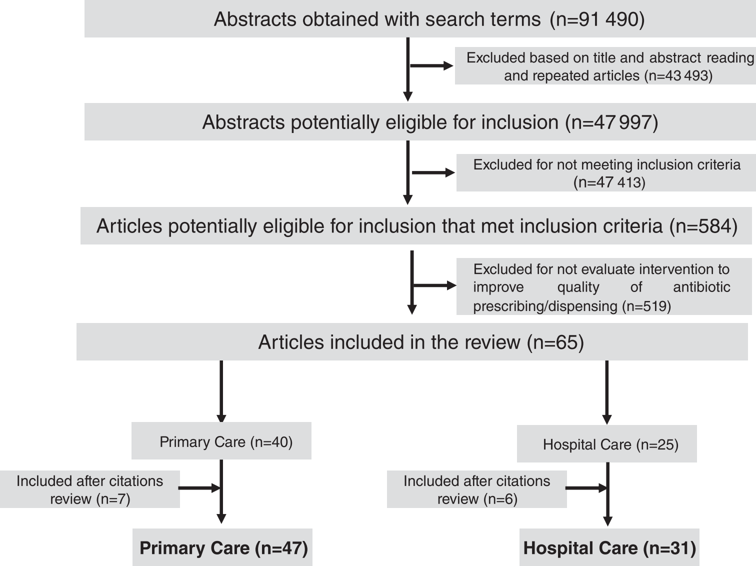
Abstracts obtained with search terms (n=91 490)
Excluded based on title and abstract reading and repeated articles (n=43 493)
Abstracts potentially eligible for inclusion (n=47 997)
Excluded for not meeting inclusion criteria (n=47 413)
Articles potentially eligible for inclusion that met inclusion criteria (n=584)
Excluded for not evaluate intervention to improve quality of antibiotic prescribing/dispensing (n=519)
Articles included in the review (n=65)
Primary Care (n=40)
Hospital Care (n=25)
Included after citations review (n=7)
Included after citations review (n=6)
Primary Care (n=47)
Hospital Care (n=31)
